# Antioxidant metabolism in galls due to the extended phenotypes of the associated organisms

**DOI:** 10.1371/journal.pone.0205364

**Published:** 2018-10-22

**Authors:** Bruno G. Ferreira, Denis C. Oliveira, Ana S. F. P. Moreira, Ana P. Faria, Lubia M. Guedes, Marcel G. C. França, Rafael Álvarez, Rosy M. S. Isaias

**Affiliations:** 1 Departamento de Botânica, Universidade Federal de Minas Gerais, Belo Horizonte, Minas Gerais, Brazil; 2 Instituto de Biologia, Universidade Federal de Uberlândia, Uberlândia, Minas Gerais, Brazil; 3 Postgrados en Ciências Biológicas - área Botánica, Univ ersidad de Concepción, Concepción, Chile; 4 Departamento de Biología Molecular– Área Biología Celular, Universidad de León, León, Spain; Institute of medical research and medicinal plant studies, CAMEROON

## Abstract

Animal-induced galls are considered extended phenotypes of their inducers, and therefore plant morphogenesis and metabolism may vary according to the species of gall inducers. The alterations in vacuolar and apoplastic polyphenols, carotenoids, chlorophyll fluorescence rates, PSII quantum yield, and phospholipid peroxidation were studied in galls induced by *Ditylenchus gallaeformans* (Nematoda) on *Miconia albicans* and *M*. *ibaguensis* (Melastomataceae), and by an unidentified Eriophyidae (Acarina) on *M*. *ibaguensis*. The focus currently addressed is gall metabolism as the extended phenotype of the gall inducers, and the neglected determination of gall functionalities over host plant peculiarities. Galls induced by *D*. *gallaeformans* on *M*. *albicans* and by the Eriophyidae on *M*. *ibaguensis* have increased accumulation of apoplastic and vacuolar phenolics, which is related to the control of phospholipid peroxidation and photoprotection. The galls induced by *D*. *gallaeformans* on *M*. *ibaguensis* have higher carotenoid and vacuolar polyphenol contents, which are related to excessive sunlight energy dissipation as heat, and photoprotection. Accordingly, antioxidant strategies varied according to the gall-inducing species and to the host plant species. The distinctive investments in carotenoid and/or in polyphenol concentrations in the studied galls seemed to be peculiar mechanisms to maintain oxidative homeostasis. These mechanisms were determined both by the stimuli of the gall-inducing organism and by the intrinsic physiological features of the host plant species. Therefore, the roles of both associated organisms in host plant-galling organisms systems over gall metabolism is attested.

## Introduction

Galls are neoformed structures induced by specific parasites on their host plants [1]. As animal-induced galls are species-specific, they are considered extended phenotypes of the galling organisms, which are supposed to control gall morphogenesis and metabolism [2–3]. However, host plant growth and metabolism are altered under the influence of the associated parasites [4–5], e.g., leading to an increase in phenolic compound contents in several insect galls [6–10]. The increment of polyphenol contents has been traditionally related to the defense against natural enemies of the galling organisms [7–9]. However, phenolics in insect galls have also been related to an increase of IAA (indol-3-acetic acid), influencing processes of cell hypertrophy and hyperplasia [6][11–13]. They are also involved in the antioxidant system, preventing the premature senescence of gall tissues [4–5,14–15].

Despite the high oxidative stress related to the greater growth and respiration rates in gall tissues, some green galls can maintain photosynthetic metabolism [13–19], which may have an important role in the maintenance of gall tissue homeostasis [4], and avoidance of hypercarbia and hypoxia [14,20]. Chloroplasts are sites of major production of reactive oxygen species (ROS), and during stressful conditions, the rates of absorbed light exceed those required for photochemical reactions, which increases ROS production and oxidative damages [21–22]. Oxidative stress then causes alterations in chlorophyll fluorescence rates, such as reduction in maximum PSII quantum yield (*F*_v_*/F*_m_), and instantaneous fluorescence decline ratio in light (R_fd_) [23–25]. PSII quantum yield is a measurement of the PSII performance, indicating the proportion of energy absorbed by chlorophylls that is directed to photochemical reactions [24], and therefore the efficiency of light energy conversion in chemical potential. During stress conditions, free radicals oxidize chlorophyll molecules, reducing PSII efficiency [23], and making PSII quantum yield an indicator of stress conditions in plants. Instantaneous fluorescence decline ratio in light (R_fd_) is an empiric parameter used to assess plant vitality, and indicates the speed of adaptation of the PSII to the exposition to saturating light. R_fd_ values are lower in stressed plant cells, in which a longer time is necessary to PSII reach the maximum capacity of energy transference to photochemical reactions, due to damages in the photosynthetic apparatus (such as chlorophylls oxidized by ROS) [14,26].

The conversion of sunlight energy into chemical potential by PSII (photochemical reactions), the chlorophyll fluorescence, and the energy dissipation as heat, promoted by carotenoids, are important mechanisms of light energy dissipation in plants [23], avoiding the oxidation of plant cell molecules. Dissipation of excessive light energy by heat is measured by non-photochemical quenching (NPQ), which indicates the ability of cell machinery to dissipate light energy by mechanisms other than photochemical reactions [23]. In insect-induced galls, increased carotenoids may help the maintenance or increment of NPQ rates, and therefore it may be considered a relative stress tolerance response, redirecting the excessive energy unabsorbed by oxidized PSII to heat dissipation [14]. Plant organs in conditions of increased oxidative stress may have temporarily (photoinhibition) or permanently oxidized chlorophyll molecules (photodamage). Consequently, a decreased capability of dissipating light energy by photochemical reactions or by chlorophyll fluorescence is expected in PSII, leading to an oxidative burst and causing major damages [21,24].

Oxidative stress is related to the peroxidation of membrane phospholipids and oxidation of proteins, which may affect cell physiology and culminate in programmed cell death (PCD) [22,25,27]. Accordingly, the primary roles of polyphenols may involve dissipation of oxidative stress induced both by abiotic and biotic factors [28], while the anti-herbivore role could be secondary [12,29], and effective just for the attack of generalists [30].

**Fig 1:**
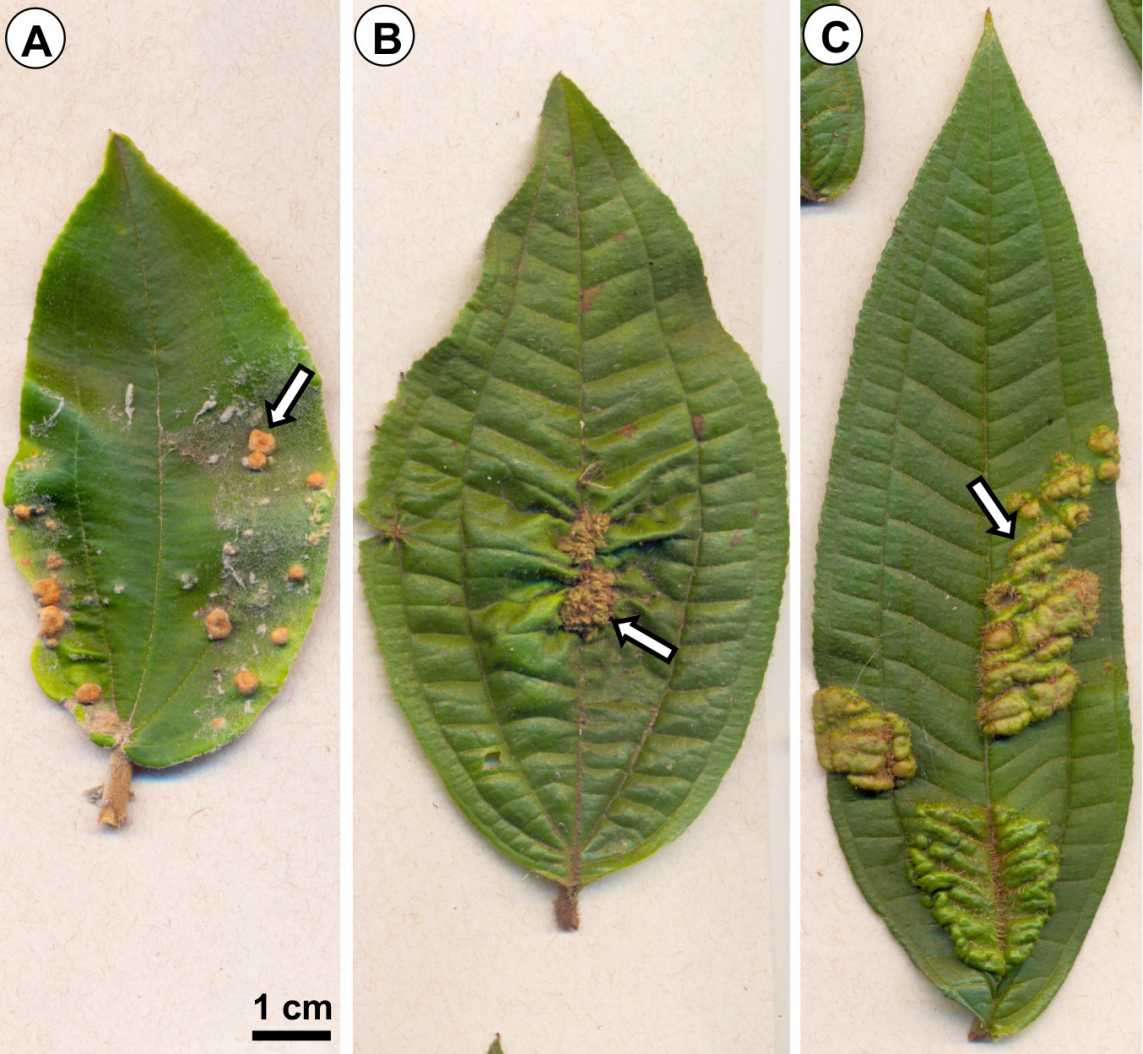
Galls studied. (A) Gall (arrow) induced by *Ditylenchus gallaeformans* (Nematoda) on a *Miconia albicans* (Melastomataceae) leaf. (B) Gall (arrow) induced by *D*. *gallaeformans* on a *M*. *ibaguensis* leaf. (C) Gall (arrow) induced by an Eriophyidae on a *M*. *ibaguensis* leaf.

Current work investigates photosynthetic and biochemical alterations in three leaf gall systems in comparison to non-galled leaves of host plants (controls). Two of them are brownish globoid verrucous galls induced by *Ditylenchus gallaeformans* Oliveira et al. 2013 (Nematoda: Anguinidae) on leaf midribs and secondary veins of *Miconia albicans* (Sw.) DC. (Fig 1A) and *M*. *ibaguensis* (Bonpl.) Triana (Fig 1B). Another gall is induced by an unidentified Eriophyidae (Acarina) species on *M*. *ibaguensis* (Fig 1C), and it is characterized by little modifications in non-galled leaves, mainly increment in pubescence on abaxial region (or more rarely on adaxial region, depending on where the mite colony installs) and leaf mesophyll homogenization [31]. The galls induced by *D*. *gallaeformans* on *M*. *albicans* and *M*. *ibaguensis* are induced on leaf primordia, and hyperplasia and hypertrophy of parenchyma mesophyll cells are stimulated. Vascularized emergences of ground and vascular tissues emerge in adaxial or abaxial surfaces, covering the colony of nematodes by involving them in a chamber closed by the curved emergence and dense hairiness [31,32]. In contact with the colonies, plant cells are maintained promeristematic, capable of continuously differentiating into new emergences toward the gall chamber [31,32]. This phenomenom indicates an indeterminate-growth capability in these nematode galls, which was not described in any other gall system [31,32]. In comparison, galls induced by the eriophyids on *M*. *ibaguensis* are structurally simple galls, mainly constituted by a dense indumentum. Eriophyidae–*M*. *ibaguensis* galls have determinate growth, and the dense indumentum is formed by non-vascularized emergences (emergences are appendages on plant surfaces formed by dermal and ground tissue systems, and occasionally vascular system). The epidermal cells are the sites of the mites feeding [31].

Currently, we compare the metabolism of galls under the influence of distinct host plants and distinct gall-inducing species. We expect that the increase of polyphenol contents should be related to the control of oxidative stress and to the maintenance of a basal photosynthetic metabolism in tissues of leaf galls induced by nematodes and mites. The comparison of distinct responses of related taxa of host plants, *M*. *albicans* and *M*. *ibaguensis*, to the same parasite, *D*. *gallaeformans*, and of same host plant, *M*. *ibaguensis*, to distinct galling parasites, *D*. *gallaeformans* and the Eriophyidae, allowed us to access if the peculiarities of gall metabolism are only extended phenotypes of the gall inducers or to what extent they can be also determined by the intrinsic host plant metabolism features.

## Methods

### Collections

Samples of *M*. *albicans* and *M*. *ibaguensis* (Melastomataceae) were collected from populations located at the Ecological Station (EEco) of the Universidade Federal de Minas Gerais (UFMG), in Belo Horizonte, Brazil (19°52’29” S; 43°58’21” W; 886 m). Leaves galled by *D*. *gallaeformans* (Nematoda: Anguinidae) on both *Miconia* spp., and the control-leaves, i.e., the correspondent opposite non-galled leaves of the same pair of leaves, were collected. The control-leaves and leaves galled by an unidentified Eriophyidae (Acarina) on *M*. *ibaguensis* (Melastomataceae) were collected in a population located near the Center of Didactic Activities 1 (CAD-1) of UFMG (19°51’56” S; 43°57’57” W; 854 m). As detailed below, for some analyses, the galled leaves were divided into non-galled portion of galled leaves (NGP), corresponding to the non-affected portions of the galled leaves; non-galled portion close to gall edge (NGCG), corresponding to the non-galled portion of galled leaves in contact with the galls (0.5 cm around the galls); and galls (the infested portions).

### Impacts on leaf area

Control (mature) leaves and galled leaves (with mature galls) (10 per sample) were collected (n = 10 individuals), scanned with a scale bar, and their areas measured with the AxioVision® software. The affected area (sum of the areas with galls) of the galled leaves was measured, and the mean percentage of affected leaf area was calculated as the affected leaf area/total leaf area.

### Relative water content

Discs (26.75 mm^2^ each) of control-leaves and galls (10 per sample) were cut (n = 10 individuals), and immediately weighted for the determination of fresh weight (FW). The samples were immersed in distilled water for 24h at 4 ^o^C, dried with a towel paper, and weighted one more time for the determination of turgid weight (TW). Then, the samples were dried in a 60 ^o^C stove for 24h and 48h, and weighted for the determination of dry weight (DW). Relative water content (RWC) was obtained following the relations (FW—DW)/(TW—DW) [33].

### Chlorophyll and carotenoid contents

Chloroplast pigments were extracted using dimethyl sulfoxide (DMSO) [34]. Leaf discs (3 per sample) (26.75 mm^2^ each) of control-leaves and galls were collected (n = 10 individuals), weighted and immersed in 5 mL of DMSO in amber bottles until complete extraction (about 24h). DMSO was added to the initial solution, reaching 10 mL of total volume, and the solution was analyzed in the spectrophotometer using triplicates, under the following wavelengths: 480 nm, 649 nm and 665 nm. Chlorophyll *a* and *b* and carotenoid contents were calculated according to Wellburn [35] and were expressed in μg mg^-1^ of fresh mass.

### Chlorophyll fluorescence

Control and galled leaves (3 per sample) were collected (n = 5 individuals), maintained in the dark for 1h, and taken to the Laboratory of Plant Anatomy for analyses in the Handy FluorCam FC 1000-H / Photon Systems Instruments®. Different photosynthetic parameters were measured for comparisons among controls and galled leaves: minimum fluorescence in dark-adapted state (*F*_0_), maximum fluorescence in dark-adapted state (*F*_*m*_), maximum PSII quantum yield in the dark-adapted state (*F*_v_*/F*_m_; where *F*_v_ = *F*_m_–*F*_0_,), PSII operating efficiency [(*F*′_m_–*F*′)/*F*′_m_]; where *F*′_m_ is the fluorescence signal when all PSII centers are closed in the light-adapted state and *F′* is the measurement of the light-adapted fluorescence signal, instantaneous fluorescence decline ratio in light (R_fd_) and steady-state non-photochemical quenching (NPQ) [(*F*_m_–*F*′_m_)/*F*′_m_] [36]. The parameters were measured on the control leaves, non-galled portions of galled leaves (NGP), non-galled portions up to 0.5 cm of the gall edge (NGCG), and on galls induced by nematodes and eriophyids.

### Histochemical analyses

To detect the accumulation of phenolics, fragments of control-leaves and of mature galls were fixed in 2.5% glutaraldehyde and 4.5% formaldehyde in phosphate buffer (0.1 M; pH 7.2) [37], dehydrated in buthanol series, embedded in Paraplast, and sectioned in rotary microtome (12 μm) [38]. The slides were deparaffinized with butyl acetate, hydrated in an ethanol series, and stained with 10% aqueous Iron(III) chloride [38].

For detection of the sites of accumulation of hydrogen peroxide (H_2_O_2_), hand-made transverse sections of fresh galls and control-leaves were submitted to 0.5% DAB (3,3-diaminobenzidine) for 15 and 30 min in the dark [39], and analyzed under a light microscope Leica ICC50 HP (Leica, Wetzlar, Germany). The images were compared to detect the main sites of peroxidase activity.

### Levels of lipid peroxidation

Control-leaves, NGP and galls (3 per sample) were collected (n = 5 individuals) at the end of rainy season (March-April), and immediately immersed in liquid nitrogen (N_2_). The material was taken to laboratory, and 0.2 g of each sample was macerated in liquid N_2_ using 20 mg of PVPP (polyvinylpolypyrrolidone). An aliquot (1 mL) of 0.01% butylated hydroxytoluene (BHT) (w/v) in 80% ethanol was added to each sample. The homogenate was centrifuged at 3,000 g for 10 min, and the supernatant was collected and maintained at 4 ^o^C. The supernatant (25 μl) was added to 25 μL of methanol, and other 25 μL was separately added to 25 μL of 10 mM TFF (triphenylphosphine) in methanol, in order to eliminate the background from other substances that could overestimate the results. The mixture was homogenized and stored at room temperature for 30 min. An aliquot of FOX reagent (1 mL) (90% methanol, 110 mM HClO_4_, 4 mM BHT, 2 mM ammonium iron(II) sulfate, and 150 μM xylenol disodium salt orange) was added to each sample, the mixtures were incubated for 10 min, and therefore they were read in quadruplicates in microplate reader at 560 nm ([27], modified). The phospholipid hydroperoxides were quantified (nmol g^-1^ of dry mass), according to equivalents of hydrogen peroxide (H_2_O_2_) (Merck) in the concentrations of 0 to 320 μm.

### Quantification of phenolics

Control-leaves, galls, and NGP were collected (5 per sample from n = 5 individuals), at the end of rainy season, and immediately immersed in liquid N_2_. The material was taken to the Laboratory of Plant Anatomy, and 0.1 g of each sample was macerated in liquid N_2_. An aliquot of 500 μL of methanol at 4^o^ C was added to each sample, the samples were vortexed, and centrifuged at 12,000 g for 5 min. The supernatant was collected and placed in clean tubes. The whole process was repeated two times with 250 μL of absolute methanol each. The extract was used to quantify the soluble phenols. An aliquote (250 μL) of 2M NaOH was added to the remaining pellet of the methanolic extraction, and maintained at 70 ^o^C for 16 h. Then, 250 μL of 2M HCl was added to each tube, which was centrifuged at 12,000 g for 5 min. The supernatants were used to quantify the phenolics associated to cell walls. The extracts of soluble and cell wall-associated phenolics were separately diluted in water (20 μL of solution + 980 μL of distilled water, 1:50, of *M*. *ibaguensis* samples; and 5 μL of solution + 995 μL of distilled water, 1:200, of *M*. *albicans* samples), and 100 μL of Folin-Ciocalteau reagent was added to each sample. After 5 min, 600 μL of a saturated solution of Na_2_CO_3_ in 1M NaOH solution was added. The samples were incubated for 1–24 h, and therefore they were read in quadruplicates in a microplate reader at 725 nm, using a chlorogenic acid standard-curve with concentrations from 0 to 250 μM. After calculation of ε = A/C, where A was the mean absorbance of chlorogenic acid curve, and C was the mean concentration of chlorogenic acid used in the calculation of the curve, the polyphenol concentration (c) of each sample was calculated in mg g^-1^ of fresh mass, by the equation c = [(A/ε) (DF/fresh mass)], where DF is the dilution factor, i.e., DF = 50 for *M*. *ibaguensis* samples, and DF = 200 for *M*. *albicans*. The concentrations in mg g^-1^ of dry mass were given after simultaneous calculation of correspondent dry mass for each category. The extractions and calculations were performed according to Gurr et al. [40].

### Statistical analyses

The means were compared using Student’s t test, or Analyses of Variance (ANOVA) followed by Tukey’s post-test in the software SigmaStat®. The data in percentage were transformed by logit linear transformation [41] before statistical comparisons. For normality and homoscedastic conditions, some data were transformed using log10. When data did not satisfy these presupposes, they were analyzed by Kruskall-Wallis test followed by Dunn’s post-test, considering *P* < 0.05. Mean and standard deviations of (gall value—control value)/(control value) obtained from each individual were used to calculate the proportional changes of the measured parameters from the control-leaves to the galls.

## Results

### Leaf area impacted by galls

**Fig 2:**
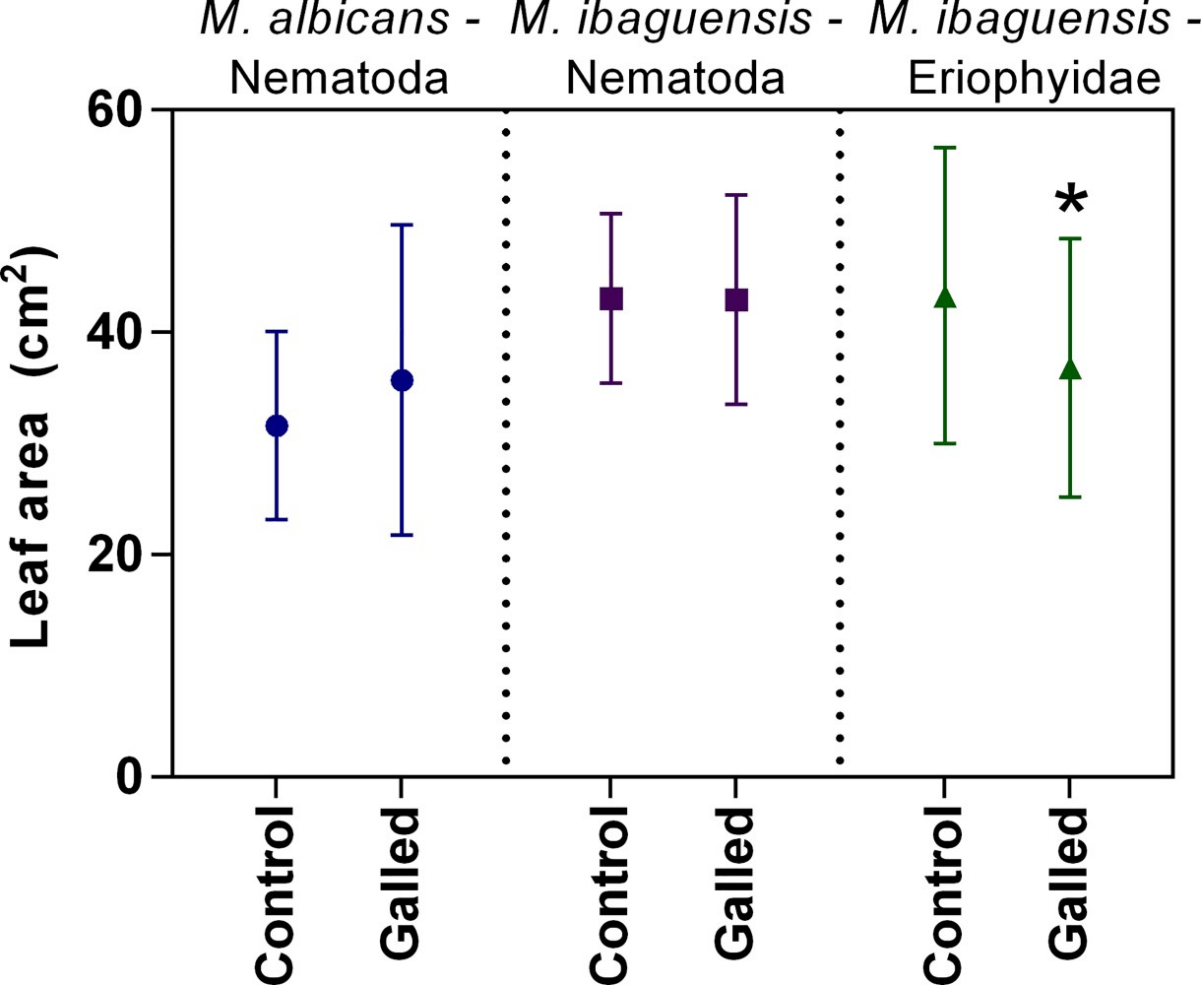
Areas (cm^2^) of control leaves and leaves galled by *Ditylenchus gallaeformans* (Nematoda) on *Miconia albicans*; by *D*. *gallaeformans* on *M*. *ibaguensis*; and by an unidentified Eriophyidae on *M*. *ibaguensis*. The bars indicate the means and standard deviations. The asterisk indicates significant difference (*P* < 0.05).

The total leaf areas of *M*. *albicans* and *M*. *ibaguensis* were not significantly altered by *D*. *gallaeformans* galling activity (Fig 2). However, an evident reduction of approximately 15% of area was observed in leaves galled by the Eriophyidae on *M*. *ibaguensis* (Fig 2). *D*. *gallaeformans* infested an average of 1.5% (± 1.31) and 2.25% (± 2.89) of leaf areas on *M*. *ibaguensis* and *M*. *albicans*, respectively. The Eriophyidae infested an average of 22.86% (± 25.58) of *M*. *ibaguensis* leaf area.

### Chloroplast pigments and relative water contents

**Fig 3:**
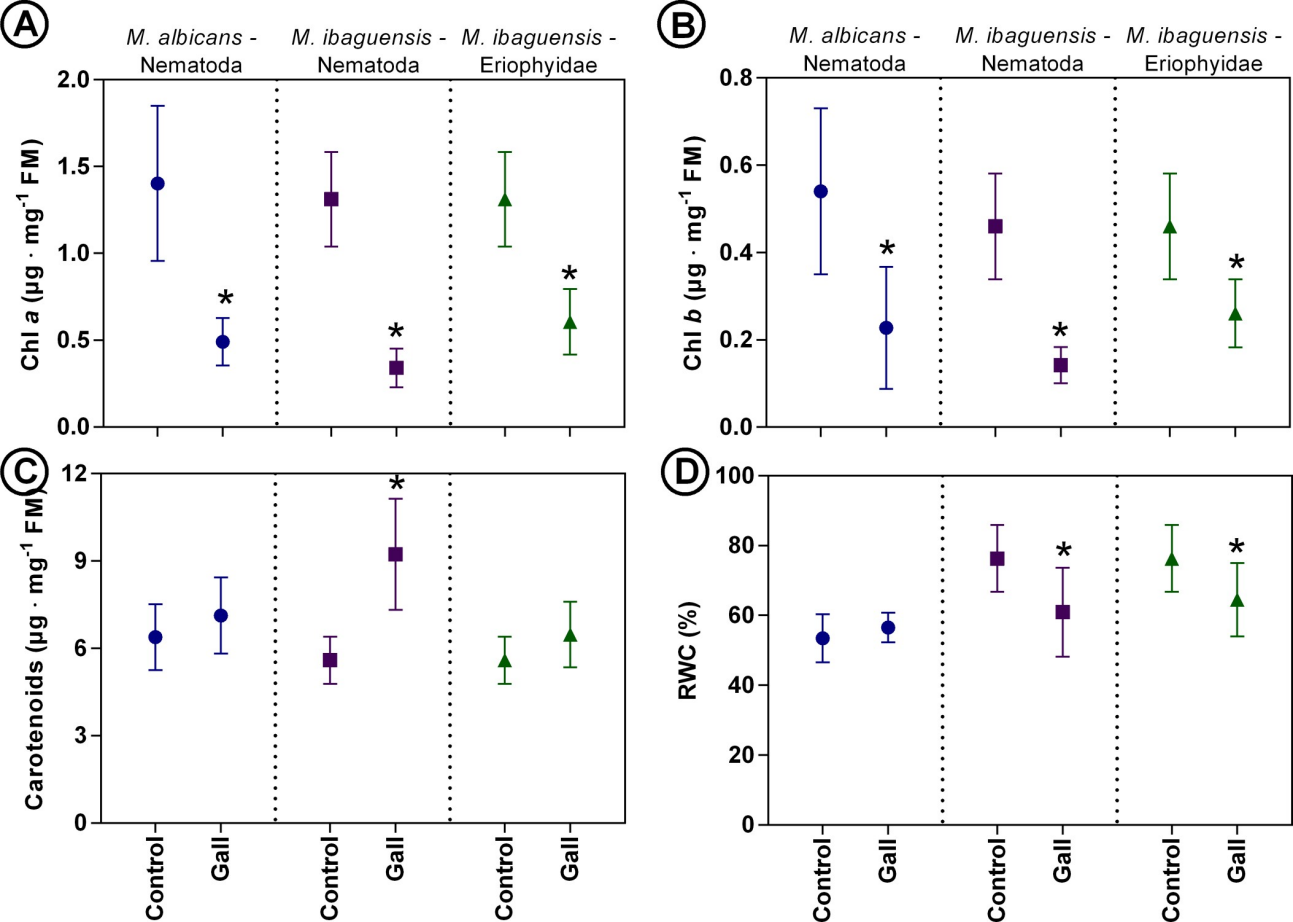
Chloroplast pigments (μg g^-1^) and relative water content (%) in leaves and galls induced by *Ditylenchus gallaeformans* (Nematoda) on *Miconia albicans*; by *D*. *gallaeformans* on *M*. *ibaguensis*; and by an unidentified Eriophyidae on *M*. *ibaguensis*. (A) Chlorophyll *a*. (B) Chlorophyll *b*. (C) Carotenoids. (D) Relative water content (RWC). Bars indicate the means and standard deviations. Asterisks indicate significant differences (*P* < 0.05).

The chlorophyll *a* and *b* contents decreased significantly in leaves galled by both gall-inducing species (Fig 3A and 3B), but the carotenoid contents did not differ in *D*. *gallaeformans*-induced galls on *M*. *albicans*, when compared to the controls. Carotenoid contents were similar between the controls and the Eriophyidae-induced galls, however, it increased approximately 65% in *D*. *gallaeformans*-induced galls on *M*. *ibaguensis* (Fig 3C). The relative water content (RWC) was similar in the controls and in the *D*. *gallaeformans* galls on *M*. *albicans*. In *M*. *ibaguensis*, the RWC was higher in the controls than in the galled leaves induced by *D*. *gallaeformans* and the Eriophyidae (Fig 3D).

### Chlorophyll fluorescence and non-photochemical quenching

**Fig 4:**
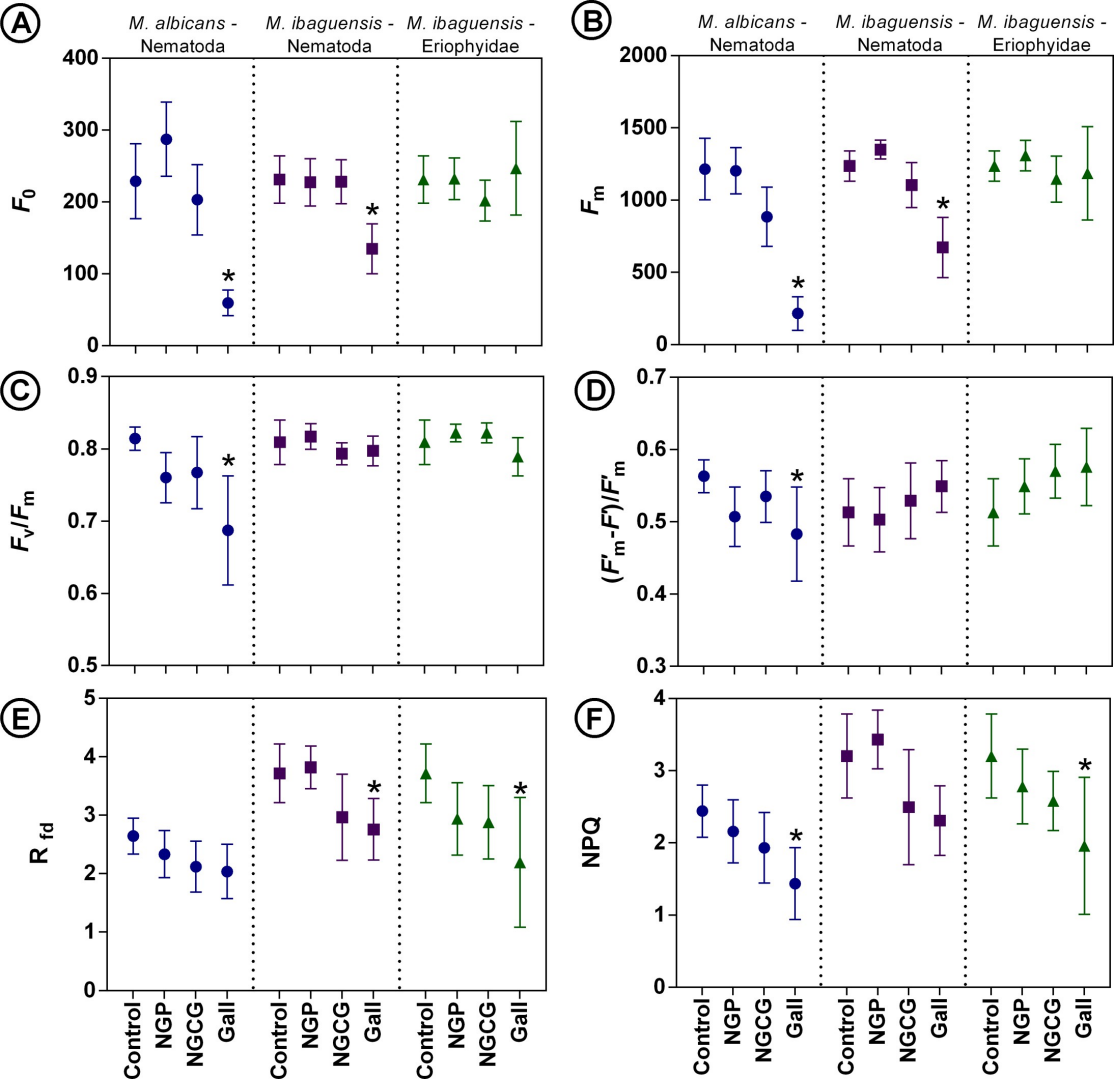
Photosynthetic parameters in leaves, non-galled portions of galled leaves (NGP), non-galled portions close to gall edge (NGCG), and galls induced by *Ditylenchus gallaeformans* (Nematoda) on *Miconia albicans*; by *D*. *gallaeformans* on *M*. *ibaguensis*; and by an unidentified Eriophyidae on *M*. *ibaguensis*. (A) F_0_ (minimum fluorescence in dark-adapted state). (B) *F*_m_ (maximum fluorescence in dark-adapted state). (C) *F*_v_*/F*_m_ (maximum quantum yield of photosystem II). (D) (*F*′_m_–*F*′)/*F*′_m_ (PSII operating efficiency). (E) R_fd_ (instantaneous fluorescence decline ratio in light). (G) NPQ (instantaneous non-photochemical quenching during light adaptation). Bars indicate the means and standard deviations. Asterisks indicate significant differences (*P* < 0.05).

The chlorophyll fluorescence rates (*F*_0_ and *F*_m_) decreased in *D*. *gallaeformans* galls, however, in the Eriophyidae galls, these rates were similar to the control leaves (Fig 4A and 4B). Also, no differences were found in the observed parameters for NGP and NGCG (Fig 4A and 4B).

The maximum PSII quantum yield (*F*_v_/*F*_m_), and the PSII operating efficiency (*F*′_m_–*F*′)/*F*′_m_ of *D*. *gallaeformans* and of the Eriophyidae galls, and respective NGP and NGCG were similar in *M*. *ibaguensis*. In *M*. *albicans*, the values of *F*_v_*/F*_m_ and (*F*′_m_–*F*′)/*F*′_m_ decreased in galls, but they did not differ in NGP and NGCG (Fig 4C and 4D).

The fluorescence decline ratio (R_fd_) did not change in *M*. *albicans* galls in relation to the control, but it decreased in *D*. *gallaeformans* and Eriophyidae galls on *M*. *ibaguensis* in comparison to the controls (Fig 4E). Even though the R_fd_ was lower in *M*. *ibaguensis* galls, it was not altered in NGP and NGCG (Fig 4E). The non-photochemical quenching (NPQ) was lower in galls induced by *D*. *gallaeformans* on *M*. *albicans*. The NPQ is also lower in the galls induced by the Eriophyidae on *M*. *ibaguensis*. No changes were observed in NPQ in *D*. *gallaeformans-*induced galls on *M*. *ibaguensis* (Fig 4F). The NPQ of NGP and NGCG was similar to the controls in all cases (Fig 4F).

### Polyphenol and ROS accumulation sites

**Fig 5:**
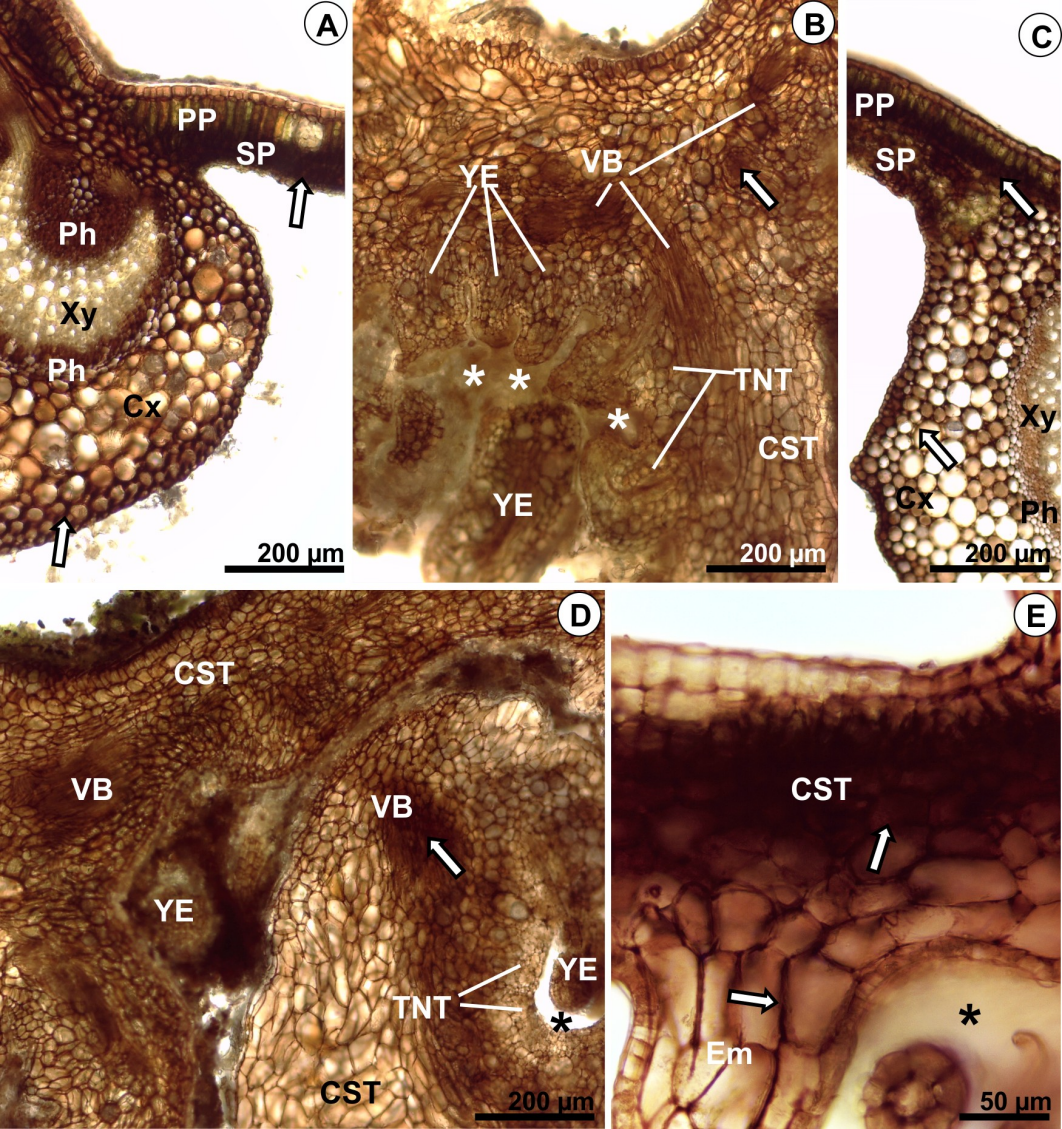
Histochemistry of hydrogen peroxide (H_2_O_2_) in leaves and galls of *Ditylenchus gallaeformans* (Nematoda) on *Miconia albicans* and *M*. *ibaguensis*, and Eriophyidae galls on *M*. *ibaguensis*. Transverse sections. The asterisks indicate the gall chambers; the arrows indicate accumulation of hydrogen peroxide. (A-B) *M*. *albicans*. (A) Midrib and mesophyll of control leaves. (B) Nematode gall. (C-F) *M*. *ibaguensis*. (C) Midrib and mesophyll of control leaves. (D) Nematode gall. (E-F) Eriophyid gall. Abbreviations: ***CST*,** common storage tissue; ***Cx*,** cortex; ***Em*,** emergence; ***Ph*,** phloem; ***PP*,** palisade parenchyma; ***SP*,** spongy parenchyma; ***TNT*,** typical nutritive tissue; ***VB*,** vascular bundle; ***Xy*,** xylem; ***YE*,** young emergences. Staining: 0.5% 3,3′-diaminobenzidine (DAB).

Hydrogen peroxide was intensely detected in palisade and spongy parenchymas, and in phloem of *M*. *albicans* control leaves (Fig 5A). In galls induced by *D*. *gallaeformans* on *M*. *albicans*, these reactive oxygen species (ROS) were intensely detected in vascular bundles, and in cell walls of common storage tissue, typical nutritive tissues, and neoformed emergences (Fig 5B). The ROS color reaction was intense in palisade and spongy parenchymas of non-galled leaves of *M*. *ibaguensis*, but moderate in midrib cortex and phloem (Fig 5C). In galls induced by *D*. *gallaeformans*, the color reaction was intense in vascular bundles and trichomes. In the common storage tissue, typical nutritive tissue, and neoformed emergences, an intense color reaction was detected in cell walls (Fig 5D). In galls induced by the Eriophyidae on *M*. *ibaguensis*, the ROS were intensely detected in common storage tissue, and in emergences (Fig 5E).

**Fig 6:**
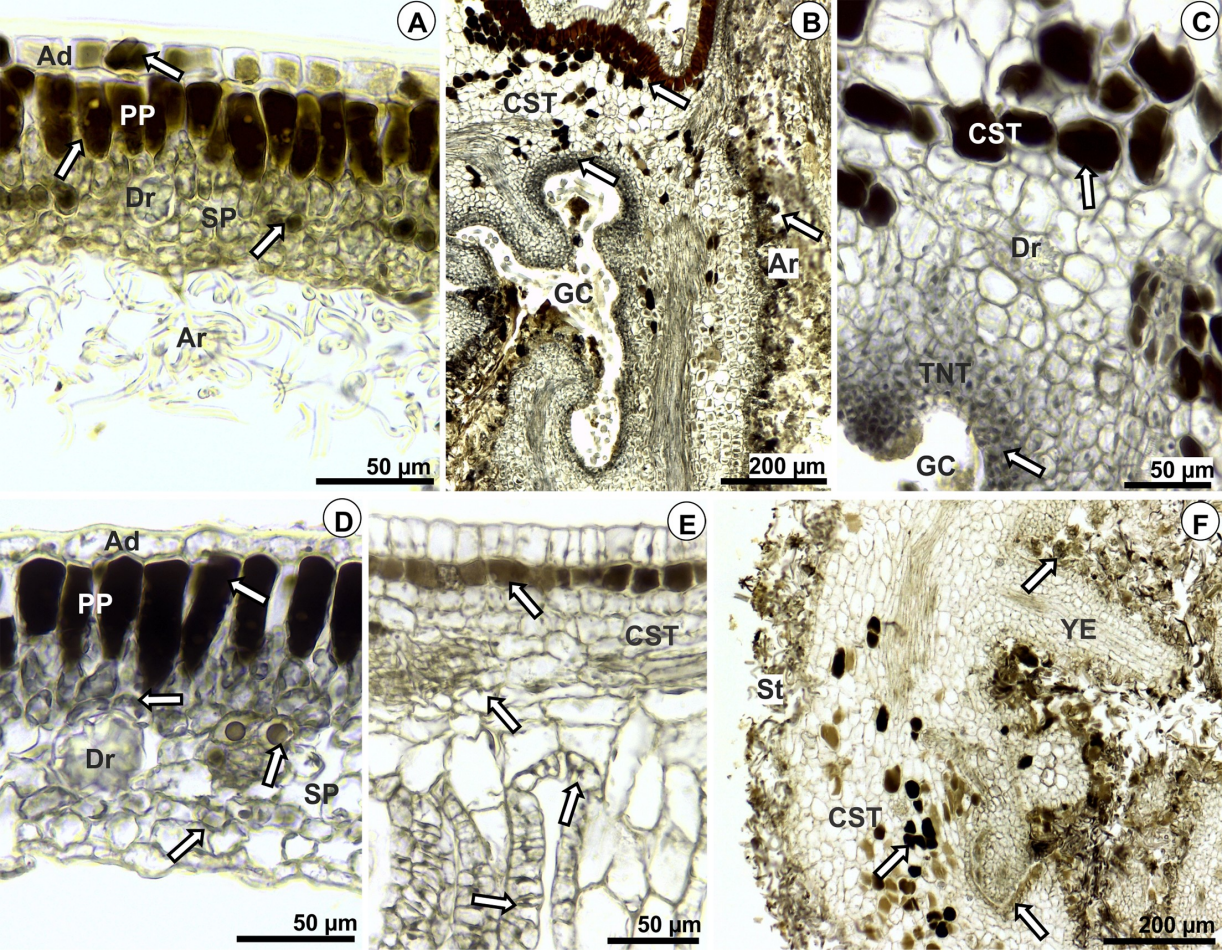
Histochemistry of polyphenols in leaves and galls induced by *Ditylenchus gallaeformans* (Nematoda) on *Miconia albicans*; by *D*. *gallaeformans* on *M*. *ibaguensis*; and by an unidentified Eriophyidae on *M*. *ibaguensis*. Transverse sections. The arrows indicate positive reaction. (A-C) *M*. *albicans*. (A) Mesophyll of control leaves. (B-C) Nematode gall. (D-F) *M*. *ibaguensis*. (D) Mesophyll of control leaves. (E) Eriophyid gall. (F) Nematode gall. Abbreviations: ***Ad*,** adaxial epidermis; ***Ar*,** arachnoid trichomes; ***CST*,** common storage tissue; ***Dr*,** druses; ***Em*,** emergence; ***GC*,** gall chamber; ***PP*,** palisade parenchyma; ***SP*,** spongy parenchyma; ***St*,** stellate trichomes; ***TNT*,** typical nutritive tissue; ***YE*,** young emergences. Staining: 10% Iron(III) Chloride.

Polyphenols were intensely detected in both epidermal surfaces and in the mesophyll of control leaves of *M*. *albicans* (Fig 6A). Polyphenols also accumulated in several layers of common storage tissue and in the trichomes of *D*. *gallaeformans* galls (Fig 6B). A moderate staining was also observed in nutritive tissues of these galls (Fig 6C). In *M*. *ibaguensis*, phenolics were intensely detected in mesophyll parenchyma and epidermal cells of control leaves (Fig 6D). Polyphenols were also detected in common storage tissue and epidermal cells of Eriophyidae galls (Fig 6E), and in the stellate trichomes, common storage tissue, and nutritive tissue of *D*. *gallaeformans* galls on *M*. *ibaguensis* (Fig 6F).

### Phospholipid peroxidation

**Fig 7:**
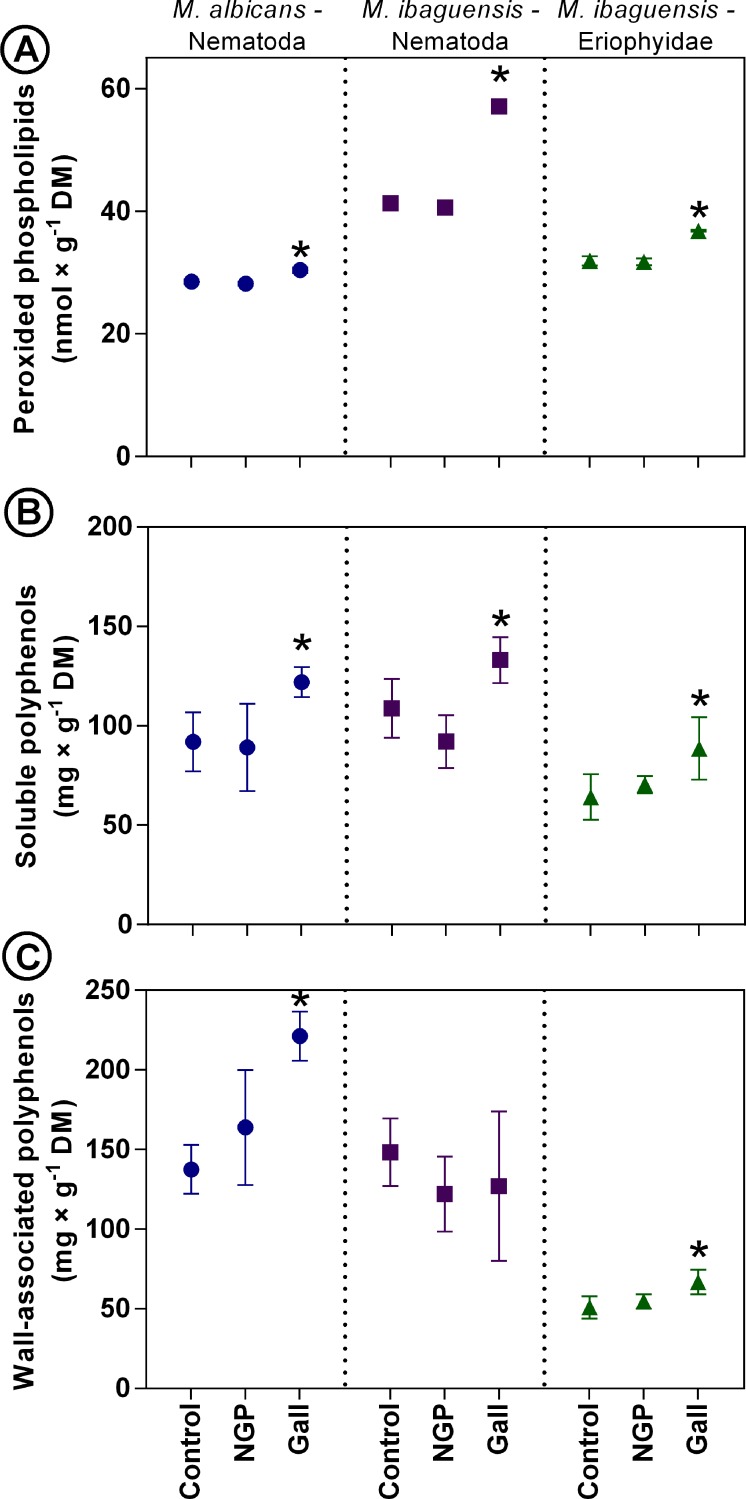
Polyphenol (mg g^-1^) and peroxided phospholipid (nmol g^-1^) contents in leaves, non-galled portions of galled leaves (NGP), and galls induced by *Ditylenchus gallaeformans* (Nematoda) on *Miconia albicans*; by *D*. *gallaeformans* on *M*. *ibaguensis*; and by an unidentified Eriophyidae on *M*. *ibaguensis*. (A) Peroxided phospholipids. (B) Soluble phenolics. (C) Cell wall associated phenolics. Bars indicate the means and standard deviations. Asterisks indicate significant differences (*P* < 0.05).

The concentrations of peroxided phospholipids were higher in all studied galls when compared to controls (Fig 7A). No differences between the control plants and the NGP were observed (Fig 7A). An increase of approximately 7% of peroxided phospholipids was observed in galls induced by *D*. *gallaeformans* on *M*. *albicans*. Peroxided phospholipids increased approximately 42% in *D*. *gallaeformans* galls on *M*. *ibaguensis*, and 15% in the Eriophyidae galls on *M*. *ibaguensis*.

### Polyphenol contents in galls

There was an increase in soluble phenolics in all galls when compared to the control leaves (Fig 7B). The soluble phenol contents increased approximately 30% in *D*. *gallaeformans* galls on *M*. *albicans* and *M*. *ibaguensis*, and 37% in the Eriophyidae galls. There were no differences between the NGP and the control leaves. The contents of cell wall-associated phenolics increased in *D*. *gallaeformans* galls on *M*. *albicans* (60%) and in the Eriophyidae galls on *M*. ibaguensis (30%), but no increasing was detected in D. gallaeformans galls on M. ibaguensis galls (Fig 7C).

### Metabolic alterations

**Fig 8:**
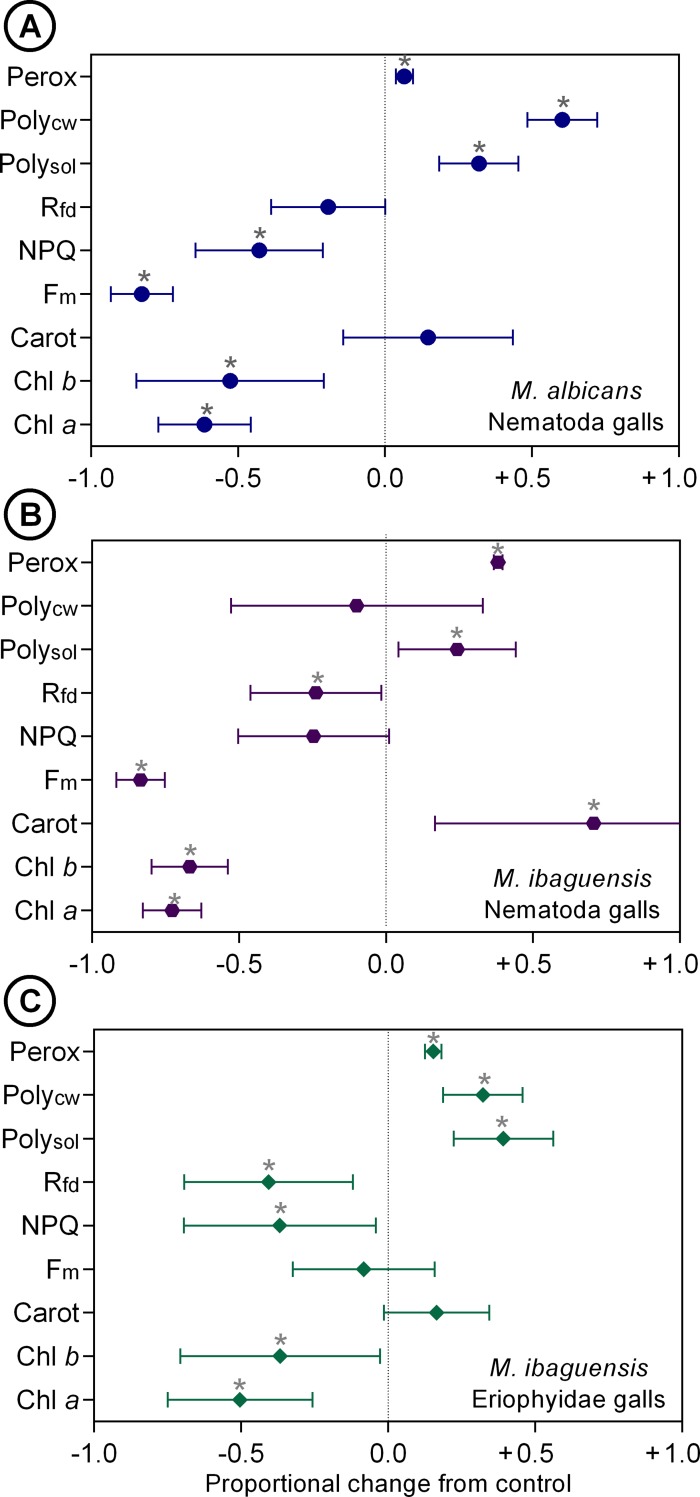
Metabolic alterations (mean and standard deviation) in galls when compared to non-galled leaves. (A) *Miconia albicans-Ditylenchus gallaeformans* (Nematoda) galls. (B) *Miconia ibaguensis-D*. *gallaeformans* galls. (C) *M*. *ibaguensis-*Eriophyidae (Acarina) galls. **Parameters: *Perox***: peroxided phospholipids; ***Poly*_*CW*_**: polyphenols associated to the cell walls; ***Poly*_*sol*_**: soluble polyphenols; ***NPQ***: non-photochemical quenching during light adaptation; ***F*_*m*_**: maximum fluorescence; ***Carot***: carotenoid content; ***Chl b***: chlorophyll *b* content; ***Chl a***: chlorophyll *a* content. Asterisks indicate statistical differences (*P* < 0.05).

Galls induced by *D*. *gallaeformans* on *M*. *albicans* leaves had the smallest alteration of phospholipid peroxidation (+7%). The maintenance of the R_fd_ occurred only in *D*. *gallaeformans* galls on *M*. *albicans* (Fig 8A). In these galls, there was an increase of soluble polyphenols and polyphenols associated to cell walls (Fig 8A). In galls induced by *D*. *gallaeformans* on *M*. *ibaguensis*, which had the greatest alteration of phospholipid peroxidation (+38%) in relation to the control leaves, the content of polyphenols associated to cell walls was maintained, but the content of soluble polyphenols increased (Fig 8B). In galls induced by the Eriophyidae on *M*. *ibaguensis*, an increase of 15% of phospholipid peroxidation was followed by an increase of soluble polyphenols and polyphenols associated to cell walls (Fig 8C).

The increase of carotenoid contents occurred only in galls induced by *D*. *gallaeformans* on *M*. *ibaguensis*, and in these galls, the NPQ was maintained (Fig 8B). In the other studied galls, the maintenance of the carotenoid contents was followed by a significant reduction of NPQ (Fig 8A and 8C). The maintenance of F_*m*_ occurred only in the Eriophyidae galls on *M*. *ibaguensis* (Fig 8C). The chlorophyll contents decreased significantly in all studied galls (Fig 8A–8C).

## Discussion

Increment of phenolics in insect-induced galls may be related to a resistance mechanism of the plants against insect attack [6–10,42]. Even though these secondary metabolites are additional protection mechanisms of plant tissues to the infection, they may consequently protect gall inducers against natural enemies such as parasitoids, inquilines, and microorganisms [8,42]. It may be also true for nematode and eriophyid galls, as those studied herein. Additionally, our hypothesis on the relation of the increment of polyphenols with the reduction of oxidative stress in gall tissues was corroborated. In plant cells, the increase in phenolic contents may be related to the enhancement of plant tissue antioxidant defenses [4–5,14,28], as observed in the three gall systems on *Miconia* spp. Apoplastic peroxidases catalyze the reaction between hydrogen peroxide and apoplastic polyphenols, by depositing phenolic polymers in cell walls, and protecting cellular membranes and important molecules against the peroxidation and the oxidation by reactive oxygen species (ROS) [43]. The lignin biosynthesis, additionally, is another metabolic process, which consumes ROS and phenolic substrates [43]. Phenolics have been proposed to avoid the irreversible oxidative damages in cellular machinery of insect galls [4–5], which may lead to cell death [44]. An increased activity of polyphenol-oxidases was related to the impairment of ROS and a major accumulation of polyphenols in insect gall tissues [15,45–47]. Herein, the reaction with DAB revealed major accumulation of ROS in cell walls, which lead us to infer the relation of polyphenols accumulation and ROS scavenging in nematode and eriophyid galls. This metabolic step is especially important in galls induced by *D*. *gallaeformans*, because of their indeterminate growth and long life cycle [31–32]. Even though *D*. *gallaeformans* galls on *M*. *albicans* and *M*. *ibaguensis* are histologically similar [31], current results demonstrate that the physiological responses of the host plants are distinct not only in response to the distinct galling herbivore species, but also regarding their own specific potentialities. Similar responses in galls induced by the same galling herbivore were expected, since it is common to consider galls as extended phenotypes of their inducers [3]. Nevertheless, we could demonstrate that the developmental and physiological responses in galls are also dependent on host plant machinery.

The ROS are supposed to be important signalizing molecules by the time of gall induction, generated by the injuries caused by the gall-inducing organisms, and by the increased respiration rates required for cell growth and proliferation [4,14,46–47]. Also, the stressful condition imposed in plant cells by excessive light energy accumulation leads to an increment of ROS production in chloroplasts [22,48]. The increment of ROS signalizes gall developmental responses and other metabolic alterations, such as the observed alterations in phenolics biosynthesis [4]. If the ROS bursts reach irreversible levels, plant cells may enter in PCD, interrupting gall establishment. Thus, the investment in antioxidant strategies would allow the maintenance of living and functional cells, and consequently of the life cycle of the galling organisms [5,45–47]. The galls of *D*. *gallaeformans* on *M*. *albicans* have the lowest increase of oxidative stress among the three gall systems studied, which is demonstrated by the minor increase in phospholipid peroxidation (+7%), and by the maintenance of fluorescence decline ratio (R_fd_). This relative stability is followed by a major production of soluble (vacuolar) polyphenols (+32%), and those associated to the cell walls (apoplastic) (+60%), indicating that the increment of apoplastic and vacuolar phenolics are in fact important antioxidant protective mechanisms in galls, as supposed for other abiotic and biotic stress conditions in plants [14,21,28,45]. The decreasing chlorophyll *a* and *b* contents are followed by a decrease in maximum (*F*_v_*/F*_m_) and operating PSII quantum yield [(*F*′_m_–*F*′)/*F*′_m_] in *D*. *gallaeformans-M*. *albicans* system. The reduction in chlorophyll contents is associated to a dilution of pigments by cell hypertrophy in some insect galls [14,17,19]. However, the similarity of the RWC between the non-galled leaves and the galls of *D*. *gallaeformans* on *M*. *albicans* indicates that the reduction of chlorophyll contents is not simply a case of pigment dilution. In these galls, the reduction in *F*_v_*/F*_m_, in operating PSII quantum yield, and in NPQ may be linked to a decrease in light energy harvesting and dissipation mechanisms [21,24,28,48]. Such processes could lead to a higher production of ROS, but an increment of apoplastic and vacuolar phenolics seems to be important to maintain the oxidative homeostasis in a more potentially photo-oxidable system. Vacuolar polyphenols are important in antioxidant dissipation responses in plants exposed to excessive sunlight energy [49]. Some polyphenols may also absorb some light wavelengths, protecting the cells against photodamage [21,28]. The antioxidant and photoprotective apparatus provided by the vacuolar and apoplastic polyphenols in *D*. *gallaeformans-M*. *albicans* galls prevents an excessive PSII oxidation. This prevention is important to support a basal rate of photochemical reactions, which has been additionally proposed to avoid hypoxia (by producing O_2_) and hypercarbia (by allowing the CO_2_ fixation reactions in the stroma of the chloroplasts) in insect gall tissues [14,20].

The physiological alterations of the galls induced by *D*. *gallaeformans* on *M*. *ibaguensis* are distinct from those of the galls induced by the same nematodes on *M*. *albicans*. In *D*. *gallaeformans-M*. *ibaguensis* system, the increase in carotenoid contents (+70%) is followed by the maintenance of NPQ, corroborating the role of carotenoids in the prevention of photodamage. Carotenoids are important in light dissipation by transference or by light conversion in heat in xanthophyll cycle [23,48]. The maintenance of NPQ may be related to the increased carotenoid content in *D*. *gallaeformans-M*. *ibaguensis* galls, revealing a distinct mechanism to avoid photodamage, when compared to the other studied galls. In fact, the increment of carotenoid/chlorophyll ratios in a stressed plant organ is important to prevent the inactivation of the PSII (photoinhibition) caused by the oxidative stress [21,48,50]. The increase in soluble polyphenols (+24%) also seems to be important to the photoprotection in these galls, as discussed above for *D*. *gallaeformans-M*. *albicans* galls. It may contribute to the maintenance of the maximum quantum yield (*F*_v_*/F*_m_) and the operating efficiency of PSII [(*F*′_m_–*F*′)/*F*′_m_]. The maintenance of a basal photosynthetic metabolism in *D*. *gallaeformans*-*M*. *ibaguensis* system is important to control the O_2_ and CO_2_ concentrations, preventing hypoxia and hypercarbia in galls by producing these molecules during photosynthetic reactions [14]. The galls of *D*. *gallaeformans* on *M*. *ibaguensis* had no significant increase in apoplastic phenolics, which may explain the higher increase in phospholipid peroxidation (+38%) and decrease in R_fd_ (-24%), when compared to other gall systems studied herein. Therefore, the apoplastic polyphenols seems to be important to the maintenance of the oxidative homeostasis in galls, where the inducer’s feeding activity and constant cell growth and replication generate an increment in ROS production [4–5].

The phospholipid peroxidation (+15%) and R_fd_ (-40%) in Eriophyidae galls on *M*. *ibaguensis* reach an intermediate level when compared to the galls of *D*. *gallaeformans*, which is also true for the accumulation of apoplastic (+39%) and soluble phenolics (+32%). As expected for the anatomically simplest of the three galls [31], the physiological impacts of the eriophyid cause a minor reduction in chlorophyll contents. The oxidative impacts are significant, but do not affect *F*_v_*/F*_m_ and PSII yield, which may be associated to the photoprotective effects of increased soluble phenolic contents [28,49]. In fact, the accumulation of vacuolar phenolics demonstrated by the histochemical reaction to Iron (III) chloride occurs mainly in the upper cell layers of the control leaves and galls. Despite of the unaffected PSII yield in Eriophyidae galls, distinct from *D*. *gallaeformans* galls, the high infestation of the Eriophyidae on *M*. *ibaguensis* causes a significant reduction in leaf area, which was observed in previously studied gall systems [51,52].

Distinct from Eriophyidae-*M*. *ibaguensis* and *D*. *gallaeformans-Miconia* spp., the effects of other galling eriophyids and some galling insects on non-galled portions of infested leaves and galls affect the fluorescence and PSII yield, which is directly related to infestation levels [53]. Therefore, *M*. *albicans* and *M*. *ibaguensis* seem to constrain the effects of the galling colonies to gall developmental sites, preventing additional oxidative damages in photosynthetic machinery. The non-alteration of phospholipid peroxidation levels and phenolic contents in non-galled portions of galled leaves may be related to strong antioxidant and photoprotective strategies of the host plants. Accordingly, the restricted impacts of the galls on *M*. *albicans* and *M*. *ibaguensis* could demonstrate the capability of these plants to control the increment of ROS in cells adjacent to gall developmental sites, due to their antioxidant systems, including the high production of polyphenols. If it is true, according to current results, plants capable of increasing polyphenol and carotenoid production during galling herbivore attack and gall development should be less affected by irreversible oxidative stress and premature cell death. On the other hand, the non-alteration in lipid peroxidation may indicate that there are no changings in oxidative stress rates in non-galled areas. Moreover, the maintenance in the sites and levels of accumulation of phenolics may also indicate a non-activation of secondary metabolism in response to the presence of the parasites.

## Conclusions

The host plant metabolic specificities determine distinct responses in the physiological impacts of the gall inducers. The responses observed in galls induced by the same parasite, *D*. *gallaeformans*, on the two host plant species, *M*. *albicans* and *M*. *ibaguensis*, reveal two strategies: first, an increase in apoplastic and vacuolar polyphenols as a regulator of oxidative homeostasis, and second, an increase of carotenoid and vacuolar polyphenol contents, which leads to the dissipation of excessive sunlight energy. Both strategies converge in maintaining a basal photosynthetic metabolism and in avoiding hypercarbia and hypoxia in gall tissues. The antioxidant role of apoplastic phenolics is the avoidance in triggering the oxidative burst in the apoplast, which may consequently prevent programmed cell death and premature gall senescence. Distinct from the common sense about the galling parasite control over its host plants, the distinct physiological responses induced by *D*. *gallaeformans* on *M*. *albicans* and *M*. *ibaguensis* demonstrate the crucial role of the host plant physiological machinery in the determination of gall phenotypes. Current results strongly indicate that galls are not strictly the extended phenotypes of their inducers, but their features reflect peculiar physiological responses of their host plants.
